# Long-segmental middle aortic coarctation: a rare case first diagnosed by transthoracic echocardiography

**DOI:** 10.1186/s12872-022-02475-2

**Published:** 2022-02-04

**Authors:** Jiwei Wang, Canying Yang, Bin Lai

**Affiliations:** 1 Department of Ultrasound, The Second Affiliated Hospital of Nanchang University, No1 Minde Road, Nanchang, 330006 Jiangxi China; 2 Department of Gastrointestinal Surgery, The Second Affiliated Hospital of Nanchang University, Nanchang, 330006 Jiangxi China

**Keywords:** Middle aortic coarctation, Middle aortic syndrome, Transthoracic echocardiography, Ultrasound, Case report

## Abstract

**Background:**

Middle aortic coarctation (MAC), also known as middle aortic syndrome, is an atypical aortic coarctation characterized by narrowing of the distal thoracic aorta and proximal abdominal aorta. MAC is a rare disease commonly diagnosed by computed tomography angiography (CTA). In this paper, we present a case of long-segmental MAC first diagnosed by transthoracic echocardiography (TTE) and further evaluated by CTA.

Case presentation.

A 14-year-old girl, with dyspnea and fatigue on exertion for 2 months and refractory hypertension for 6 months, was referred by the local clinic to our hospital. Physical examination showed blood pressure up to 176/100 mmHg measured in the arms despite dual antihypertensives, a marked pressure gradient between her arms and legs, and weak pulses in both dorsal pedes arteries. TTE revealed a segmental narrowing in the descending thoracic aorta below the level of the atrioventricular sulcus, with a calcified plaque in the stenotic region. Abdominal vascular ultrasound revealed the segmental narrowing extending to the descending abdominal aorta (5.7 mm in diameter) above the level of the superior mesenteric artery. Subsequently, CTA verified a long-segment narrowing in the descending aorta from the level of T8 to L2 vertebra, with a calcified plaque in the stenotic aorta, right renal artery involvement, and a rich network of collateral vessels between the pre-and post-stenotic region. The patient was referred for cardiovascular surgery in which a successful ascending aorta-abdominal aorta bypass was performed.

**Conclusions:**

Although MAC is usually diagnosed by CTA, it may also be first diagnosed by TTE in some patients whose longitudinal axis view of the thoracic descending aorta could be shown. Careful TTE scan can improve the diagnostic rate of MAC, especially for some hypertension patients whose marked pressure gradient between arms and legs was ignored by the physician.

**Supplementary Information:**

The online version contains supplementary material available at 10.1186/s12872-022-02475-2.

## Background

Aortic coarctation (AC) is characteristically located at the aortic isthmus which is in the proximal portion of the descending aorta, just distal to the origin of the left subclavian artery. In rare circumstances, AC is located far from the aortic isthmus which is called “atypical AC”.

Middle aortic coarctation (MAC) is a kind of atypical AC. It refers to narrowing in the middle of the aorta, typically involving the distal thoracic aorta and proximal abdominal aorta, with or without renal artery involvement [1]. MAC is a rare disease, accounting for 0.5–2% of AC [2]. Patients with MAC usually present with severe hypertension and non-specific symptoms such as headache, dyspnea and fatigue. They may also have symptoms of distal arterial insufficiency, such as lower extremity claudication and abdominal angina, but infrequent [3].

Due to the restricted accessibility of echocardiography or abdominal sonography, MAC is commonly diagnosed by computed tomography angiography (CTA)). In this paper, we present a case of MAC first diagnosed by transthoracic echocardiography(TTE) and further evaluated by CTA.

## Case presentation

A 14-year-old girl was diagnosed with hypertension in the local clinic due to dizziness and treated with Enalapril maleate (10 mg, once/day) and Cilnidipine (10 mg, once/day) for 6 months. However, her blood pressure continued to be refractory. In addition, she felt dyspnea and fatigue on exertion during the past 2 months. So, she was referred to our hospital. Physical examination showed hypertension and a marked pressure gradient between the upper and lower limbs (176/100 versus 90/50 mmHg), a grade 3/6 systolic murmur along the left sternal border and subxiphoid, and weak pulses in both dorsal pedes arteries. Therefore, AC was clinically considered. Then, she was suggested to take an electrocardiogram and TTE. The electrocardiogram showed sinus rhythm at 86 bpm with the sign of left ventricular hypertrophy and ST-T wave abnormalities.

**Fig. 1:**
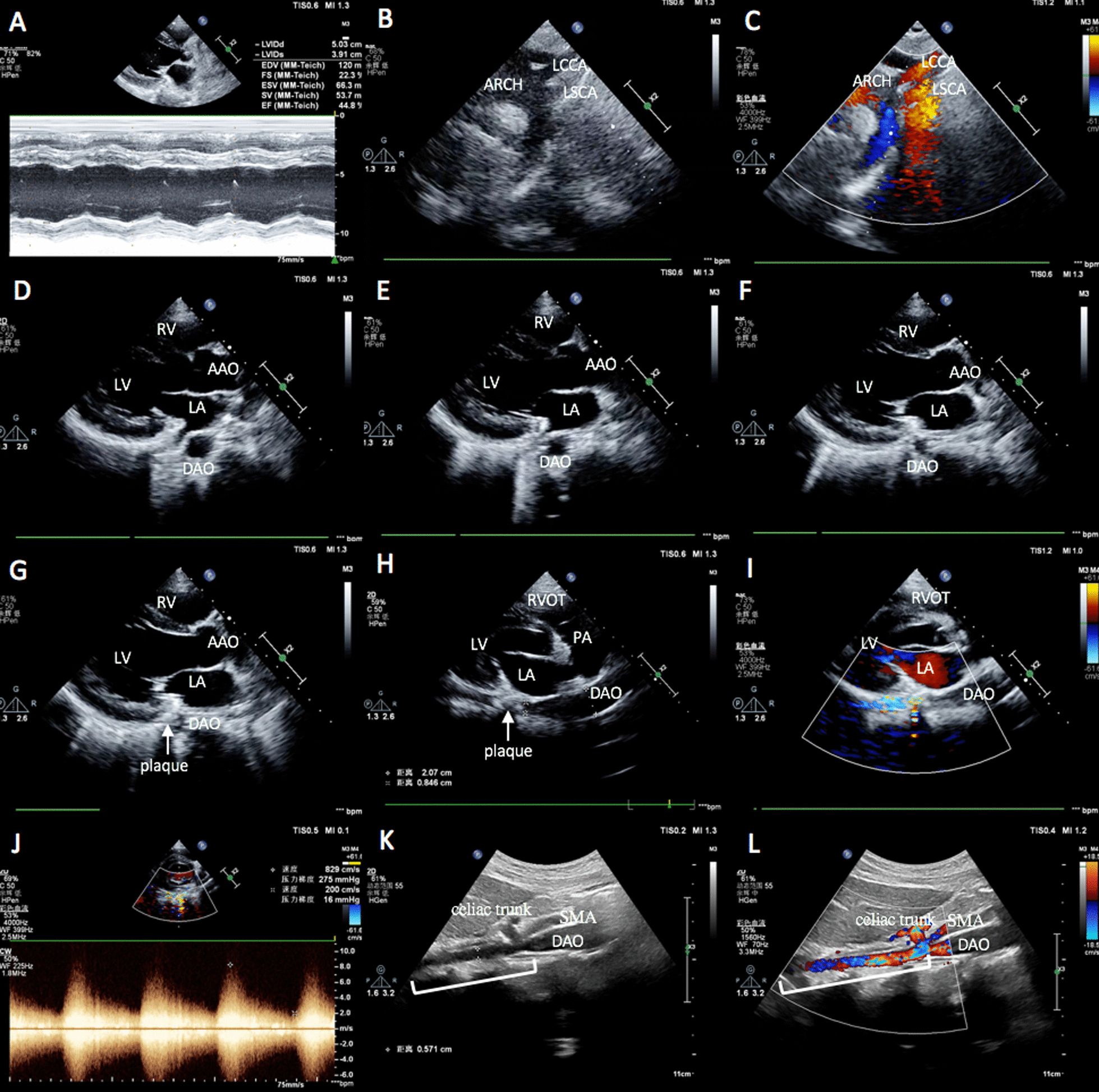
TTE and abdominal vascular ultrasound showed long segmental middle aortic coarctation. **A** M mode showed the left ventricular hypertrophy with a reduced ejection fraction to 44.8%. **B**, **C** The suprasternal view showed no aortic coarctation in the isthmus. **D**–**G** With a slight adjustment of the probe in the parasternal long-axis view of the left ventricle, the diameter of the thoracic descending aorta behind the left atrium gradually decreased. **H**–**J** The longitudinal axis of the thoracic descending aorta showed a segmental narrowing with a calcified plaque(arrow) in the thoracic descending aorta below the level of the atrioventricular sulcus, Color Doppler showed a turbulent flow in the narrowing segment with a peak velocity of 829 cm/s. **K**–**L** Abdominal vascular ultrasound showed a long segment narrowing (bracket) of descending abdominal aorta above the level of the superior mesenteric artery. *LV* left ventricle, *LA* left atrium, *RVOT* right ventricular outflow tract, *RV* right ventricle, *SMA* superior mesenteric artery, *AAO* ascending aorta, *DAO* descending aorta, *ARCH* aortic arch, *LCCA* left common carotid artery, *LSCA* left subclavian artery

The TTE showed left ventricular hypertrophy with a reduced ejection fraction to 44.8% (Fig. 1A), no coarctation in the aortic isthmus (Fig. 1B, C). However, with a slight adjustment of the probe in the parasternal long-axis view of the left ventricle, the diameter of the thoracic descending aorta behind the left atrium was inconsistent (Fig. 1D–G, Additional file 1). To show the longitudinal axis of the thoracic descending aorta, we did a non-standard view by rotating the probe clockwise about 45° from parasternal long-axis view of the left ventricle to the long axis of the body and found that a segmental narrowing in the thoracic descending aorta below the level of the atrioventricular sulcus, with a calcified plaque in the stenotic region (Fig. 1H, Additional file 2). Color Doppler showed a turbulent flow and elevated peak systolic velocities measuring up to 829 cm/s in the stenotic region (Fig. 1I, J, Additional file 3). Abdominal vascular ultrasound revealed the segmental narrowing extending to the descending abdominal aorta (5.7 mm in diameter) above the level of the superior mesenteric artery (Fig. 1K, L).

**Fig. 2:**
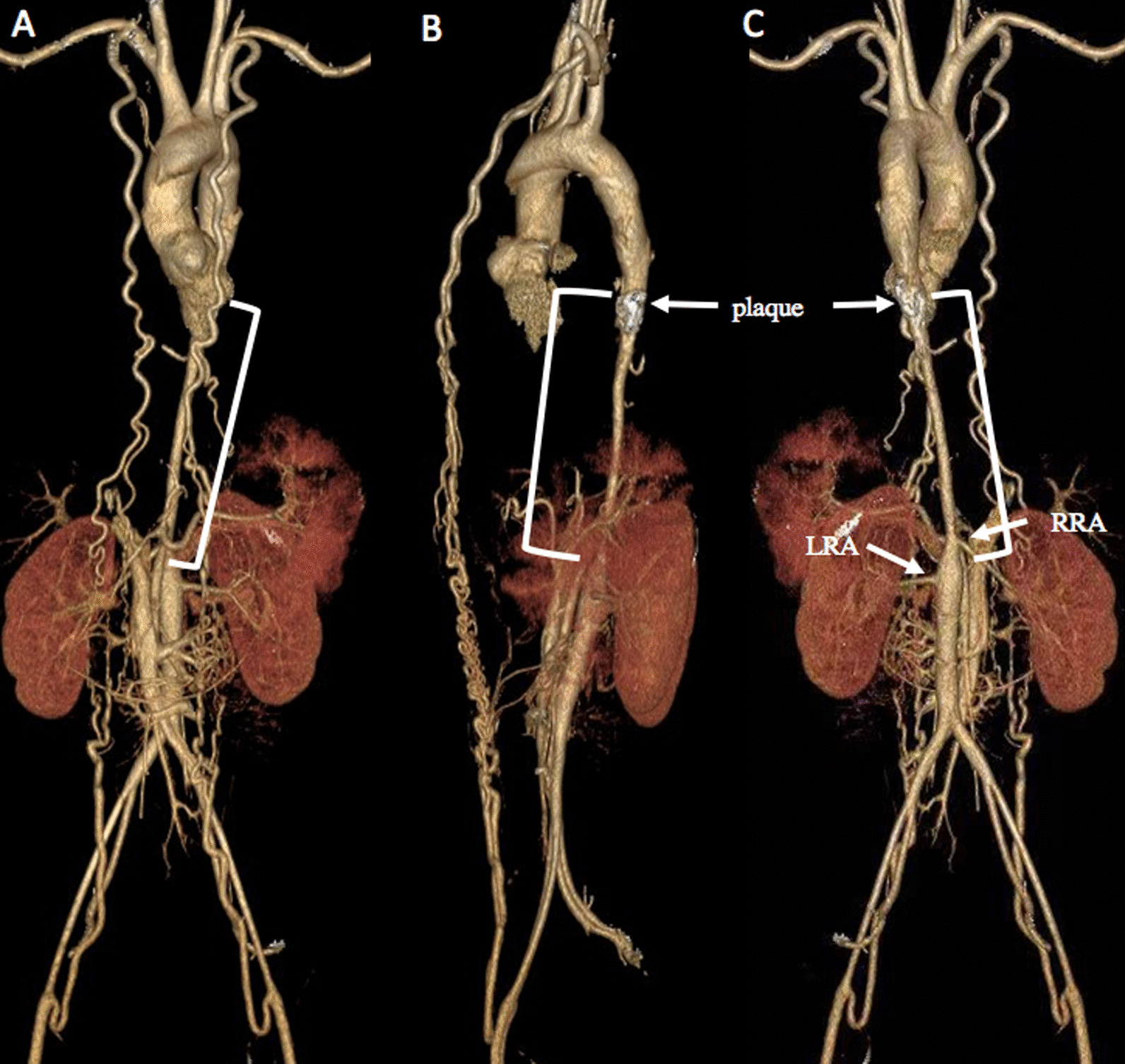
CTA imaging demonstrated a long-segment narrowing (bracket) in the descending aorta at the level of T8–L2 vertebra, with a calcified plaque in the stenotic aorta, RRA involvement and a rich network of collateral vessels between the pre-and post-stenotic region. **A** Frontal view; **B** Lateral view; **C** Posterior view. *LRA* Left renal artery, *RRA* Right renal artery

Subsequently, CTA verified a long-segment narrowing in the descending aorta from the level of T8 to L2 vertebra, with a calcified plaque in the stenotic aorta, right renal artery involvement, and a rich network of collateral vessels between the pre-and post-stenotic region (Fig. 2).

Laboratory analysis showed the complete blood count, serum electrolytes, urea, creatinine, liver function, renal function, coagulation tests and inflammatory markers were all within normal reference ranges.

Therefore, a diagnosis of MAC was made. The patient was referred for cardiovascular surgery in which a successful ascending aorta-abdominal aorta bypass was performed. One year following surgery, the patient had normal blood pressure and no symptoms without the need for medication.

## Discussion and conclusions

MAC, also known as middle aortic syndrome, is an atypical AC which usually seen in children and adolescents. It is a rare vascular disease characterized by narrowing of the distal thoracic aorta and proximal abdominal aorta.

The clinical presentations of MAC vary depending on the extent and location of coarctation [3]. Patients with MAC usually present with severe upper body arterial hypertension and non-specific symptoms such as headache, dyspnea and fatigue. Due to the gradual development of stenosis allowing the formation of effective collateral circulation pathways, symptoms of distal arterial insufficiency, such as lower extremity claudication and abdominal angina, are infrequent. The physical examination typically presents with weak or absent femoral pulses, a difference in blood pressure between arms and legs, and a systolic vascular murmur over the region of stenosis.

The etiology of MAC can be either congenital or acquired [4]. Congenital MAC has been explained by the failure of normal fusion of the two dorsal aortas. MAC may have genetic causes, such as neurofibromatosis type I (von Recklinghausen disease), Alagille syndrome, or Williams syndrome [5–8]. Acquired MAC is usually caused by inflammatory diseases or infection including Takayasu arteritis, tuberculous and rubella [9–11]. As our patient had no history of any systemic inflammatory manifestations, her inflammatory markers were within inference range, and there was no evidence of the above-mentioned genetic disorders associated with MAC. Therefore, the etiology of our patient was speculated to be congenital.

**Fig. 3:**
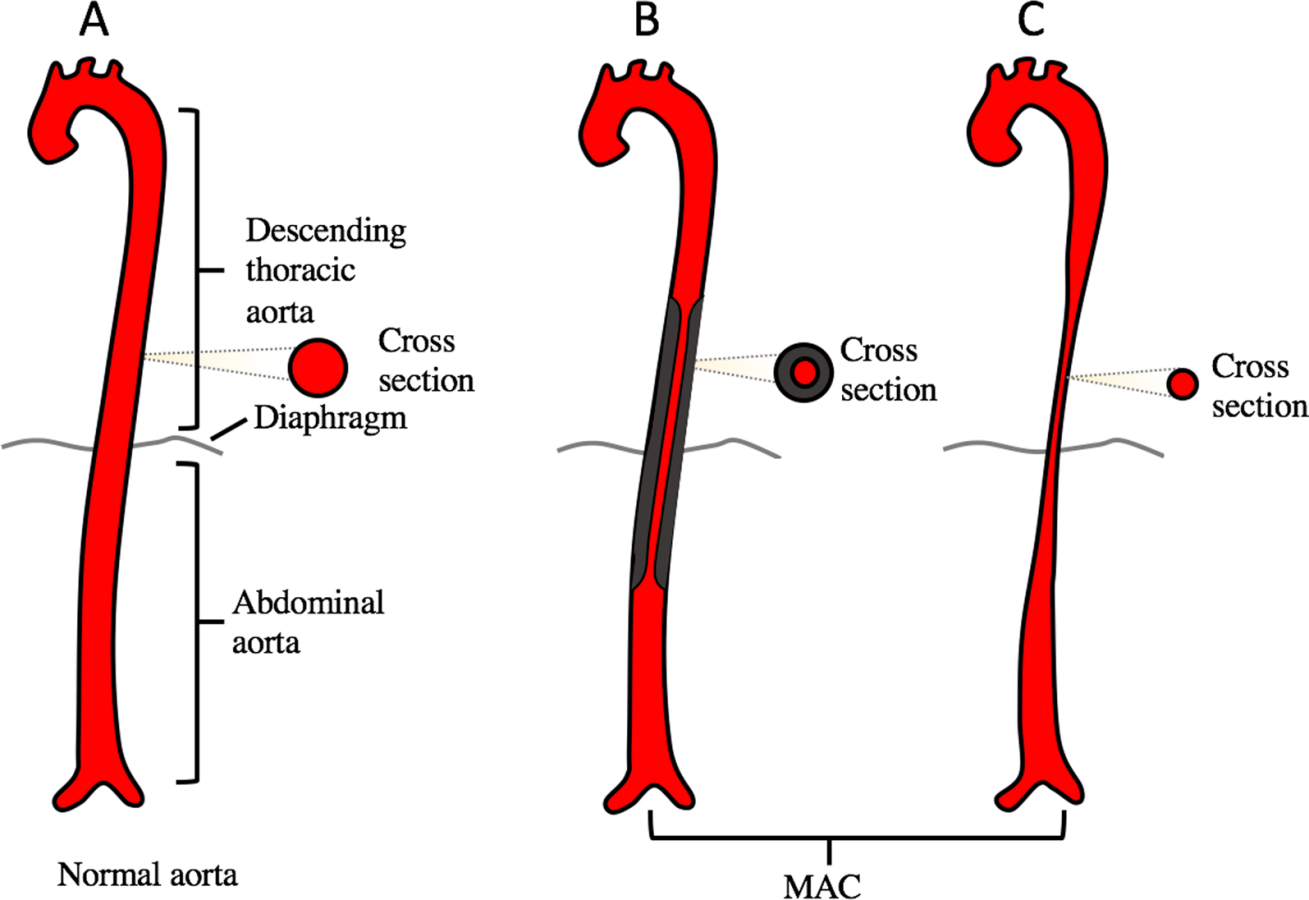
Schematic diagram of MAC. **a** Normal aorta; **b** Stenosis caused by acquired etiologies; **c** Stenosis caused by congenital dysplasia

The gold standard for the diagnosis of MAC is angiography. Diagnosis could also be made by some imaging methods, such as CTA, MRA and ultrasound [12–14]. Although ultrasound may be restricted accessibility for distal thoracic aorta in some patients, it is always the first-line imaging method to evaluate the etiology and complications of hypertension which is the main complaint of MAC patients. Therefore, careful vascular ultrasound examination of descending thoracic aorta and abdominal aorta could be taken when AC was suspected but no coarctation in the aortic isthmus. According to theirs’ different pathological manifestations (Fig. 3), ultrasound could also be used to differential diagnose the congenital and acquired MAC. For acquired MAC, it usually has evidence of an adventitial inflammatory reaction and fibrous proliferation caused by viruses or inflammatory processes. Therefore, ultrasound always shows a diffuse circumferential arterial wall thickening, resulting in a significant reduction in the inner diameter, but the outer diameter is normal. This sign is particularly typical in Takayasu arteritis, which is called the “macaroni sign” [15]. For congenital MAC, the narrowing comes from hypoplasia and there is no inflammatory reaction. So, ultrasound usually reveals that both outer and inner diameter are significantly decreased with normal intima-media thickness. As both outer and inner diameter are significantly decreased without significantly circumferential arterial wall thickening in our patient, the etiology was further speculated to be congenital.

Management of MAC is complex. Although there are a variety of treatment options available, they should be individualized. Medical treatments include antihypertensive therapy for patients with mild-to-moderate stenosis or before surgery, anti-inflammation and anti-infection for those with acquired MAC. Surgical treatments are required for patients with severe stenosis leading to refractory hypertension and targeted organ injury, which include bypass, angioplasty, or percutaneous transluminal angioplasty(PTA). However, Surgery may be attempted in infancy but is technically difficult [16, 17]. And also, PTA is not likely to achieve a favorable outcome in patients with long-segmental narrowing or hypoplasia [18].

This case highlights (1) AC should be considered during the diagnostic workup of hypertension, especially in young patients; (2) a thorough physical examination, such as marked pressure gradient between the upper and lower limbs, systolic murmur over the region of stenosis and weak femoral pulses, could raise the suspicion of AC; (3) careful vascular ultrasound examination of descending thoracic aorta and abdominal aorta should be taken when AC was suspected but no coarctation in aortic isthmus; and (4) understanding the sonographic features of MAC could be useful to differential diagnose the congenital and acquired MAC.

MAC is a rare vascular disease usually diagnosed by CTA. CTA has incomparable advantages for diagnosing MAC, especially in evaluating the extent and length of the narrowed segment and assessing the collaterals between the proximal and distal segments. However, MAC may also be first diagnosed by TTE in some patients whose longitudinal axis view of the thoracic descending aorta could be shown. Careful TTE can improve the diagnostic rate of MAC, especially for some hypertension patients whose marked pressure gradient between arms and legs was ignored by the physician.

## Supplementary Information

**Additional file 1: Video 1.** With a slight adjustment of the probe in the parasternal long-axis view of the left ventricle, the diameter of the thoracic descending aorta behind the left atrium gradually decreased.

**Additional file 2: Video 2.** The longitudinal axis of the thoracic descending aorta showed a segmental narrowing with a calcified plaque in the thoracic descending aorta below the level of the atrioventricular sulcus.

**Additional file 3: Video 3.** Color Doppler showed a turbulent flow in the narrowed thoracic descending aorta.

## Data Availability

All data generated or analysed during this study are included in this published article and its Additional files 1, 2 and 3. Additional data will be made available from the corresponding author on reasonable request.

## Abbreviations

MAC: Middle aortic coarctation
AC: Aortic coarctation
TTE: Transthoracic echocardiography
CTA: Computed tomography angiography
MRA: Magnetic resonance angiography
PTA: Percutaneous transluminal angioplasty
